# An online decision aid for patients with prostate cancer evaluating local treatment options reduces decisional conflict and distress and improves knowledge: Postmarket surveillance in German routine care

**DOI:** 10.1007/s00345-026-06368-3

**Published:** 2026-04-05

**Authors:** Philipp Karschuck, Tobias Kessler, Philipp Reimold, Luka Flegar, Gita Schönberg, Paul Schneider, Andreas Ihrig, Tanja Krones, Marco Knöll, Elke Kessler, Christian Wülfing, Maurice Stephan Michel, Christer Groeben, Johannes Huber

**Affiliations:** 1 Department of Urology, Heidelberg University Hospital, Im Neuenheimer Feld 420, DE-69120 Heidelberg, Germany; 2 Neurology and Neurooncology Program, National Center for Tumor Diseases, Heidelberg University Hospital, Heidelberg, Germany; 3 ASD Concepts GmbH & Co. KG, Institute for Patient-Centred Methods of Care, Reinheim, Germany; 4 Department of General Internal Medicine and Psychosomatic, Division of Psychooncology, University of Heidelberg, Heidelberg, Germany; 5 Institute of Biomedical Ethics and History of Medicine, University Hospital Zurich, Zurich, Switzerland; 6 Department of Urology, Asklepios Klinik Altona, Hamburg, Germany; 7 Department of Urology and Urosurgery, University Hospital Mannheim, Mannheim, Germany

**Keywords:** Decision aid, Shared decision-making, Prostate cancer, Postmarket surveillance, Health services research

## Abstract

**Background and Objective:**

Since 2016, more than 26,000 patients have used the online prostate cancer (PC) decision aid “Entscheidungshilfe Prostatakrebs”. Because a large, randomized, controlled evaluation trial failed to show effects regarding the primary and secondary outcomes after 4 weeks, we used postmarket surveillance data for further analyses. The aim of this study was to analyze the immediate effects of using a PC decision aid on decisional conflict, distress, and knowledge.

**Methods:**

A total of 2,245 patients used the PC decision aid between 05/2023 and 01/2024. We applied identical validated instruments before and after use to record immediate effects in an intra-individual comparison; the endpoints were decisional conflict (Decisional Conflict Scale, DCS), distress (Distress Thermometer), and objective knowledge (Decision Quality Worksheet).

**Results:**

Complete data were available for 880 patients (39.2%). The mean patient age was 66.3 ± 7.2 (range 43–84) years. The DCS score improved from 37.5 ± 23.7 before to 20.1 ± 15.7 after using the decision aid (p < 0.001). This effect was consistent across all the DCS subscales: level of information, clarity, support, and uncertainty. The Distress Thermometer (0: no distress; to 10: maximum distress) showed an improvement of 3.1 points from 6.2 ± 2.6 to 3.1 ± 2.6 (p < 0.001). Objective knowledge significantly improved for 4 out of the 5 questions, with differences in absolute percentages of correct answers ranging from 18.8% to 30%. The number of correct answers before vs. after using the PC decision aid increased from 2.3 ± 1.1 to 3.0 ± 1.1 (p < 0.001).

**Conclusion:**

In conjunction with the results of our previous work, we can now report strong immediate effects that diminish over a period of four weeks: the evaluated online PC decision aid reduces decisional conflict and distress and improves patient knowledge. These results provide further support for offering this tool to all suitable patients. An ongoing randomized controlled study will address the current study limitations.

**Supplementary Information:**

The online version contains supplementary material available at 10.1007/s00345-026-06368-3.

## Introduction

Patient-centered care is critical for individualized local treatment decisions in prostate cancer (PC) patients. In uro-oncology, decision aids are increasingly being investigated, but they are rarely used in routine care. These tools are designed to support patients in making good health care decisions together with their treating physician by offering assistance in conveying information and clarifying personal values [1–5]. On the basis of evidence from a recent Cochrane review, decision aids improve participant knowledge and accuracy of risk perceptions and decrease decisional conflict related to feeling uninformed, indecision about personal values, and the proportion of people who are passive in decision‐making [6]. Decision aids were analyzed for 71 different entities described across 209 studies; 80% of these studies were from English-speaking countries. Only eleven studies were focused on the treatment of PC, and overall, there are too few randomized studies on the effects of decision aids in low-risk prostate cancer patients [4].

In 2016, we described the online PC decision aid “Entscheidungshilfe Prostatakrebs” (www.entscheidungshilfe-prostatakrebs.info) in detail [7, 8]. As part of a complete makeover, we upgraded the online PC decision aid IT infrastructure and registered it as a class 1 medical device in 2021. This tool has successfully been established in routine clinical practice in Germany and can be used free of charge at www.entscheidungshilfe-prostatakrebs.de. In our recently published 1:1 randomized controlled trial (n = 1,115) comparing our PC decision aid vs. a printed information brochure, we investigated treatment decisions after 14 months as the primary outcome [7, 9]. We also measured seven secondary outcomes with validated questionnaires: decisional conflict, knowledge, acceptability, physician–patient communication, anxiety and depression, decision regret and quality of life. Patients reported follow-up data one month and 8–14 months after using the online PC decision aid. We found no significant differences in the primary or secondary outcomes [7, 9]. This lack of visible effects on the typical endpoints of shared decision-making might be attributable to measuring these aspects too late after the intervention. We are, therefore, now approaching this hypothesis with an intra-individual study design originating from our postmarket surveillance in German routine care.

The aim of this study was to analyze the immediate effects of using a PC decision aid on decisional conflict, distress, and knowledge.

## Materials and methods

The PC decision aid is an internet-based software application for patients after PC diagnosis [7, 8]. In continuous service for almost 10 years, it was the first German language personalized patient decision aid for the treatment of nonmetastatic PC. The PC decision aid offers individualized patient information, additional questionnaires for diagnostics, and preparation for a participative treatment decision [10]. The browser-based online tool includes guideline-based content [11], using 17 educational videos in the German language with a total duration of more than one hour, and is based on the newest version of the German S3 PC guidelines on. This PC decision aid has been developed to support the decision-making process, but the final decision on treatment remains with the patient and his urologist. The tool should be used as an integral part of the doctor–patient consultation: The patient receives an access card on which his or her urologist enters the following key clinical parameters to ensure correct personalization: month of initial diagnosis, initial PSA value, clinical TNM stage, Gleason score, proportion of positive prostate biopsy cores, and maximum tumor involvement of positive cores. While using the decision aid, the patient can enter all relevant personal and medical information. This input is used to personalize the videos according to three strata (oncologic risk, life expectancy, and erectile function) and to create a one-page summary as a basis for the following physician consultation [12]. Urologists can order patient access cards for free at bestellung@entscheidungshilfe-prostatakrebs.de. Patients can register themselves at www.entscheidungshilfe-prostatakrebs.de using the information on the patient access card provided by their urologist. Online Supplement 1 provides an overview of the PC decision aid and explains the study design in more detail.

We previously reported the evaluation data of 11,290 patients and 91 urologists [7]. Overall, as of October 2025, more than 26,000 patients have consulted the PC decision aid in German routine care [7]. A patient representative of the Prostate Cancer Patient Support Organization of Germany (BPS) is part of the project team. The manufacturer of the decision aid and owner of the CE mark is ASD Concepts GmbH & Co. KG (Reinheim, Germany). In 2021, the German Society of Urology (DGU; www.urologenportal.de) founded its subsidiary “Urologische Stiftung Gesundheit gGmbH” (USG; www. urologische-stiftung-gesundheit.de). Thereafter, the DGU assigned further project development to the USG.

### Study course

As part of the postmarket surveillance for a class 1 medical device (MDD), we analyzed patient usage of the PC decision aid from 05/2023 to 01/2024. This evaluation fulfills the requirements of the regular evaluation specified in Art. 83, 84 and 85 of the Medical Devices Act. We examined routine usage data, including patient-reported basic sociodemographic information supplemented with identical instruments *before* and *after* PC decision aid use. This enabled intra-individual comparisons to measure the immediate effects of using the PC decision aid.

### Validated instruments for decisional conflict, distress, and knowledge

We measured three outcomes with validated instruments, which were also applied in our earlier work [9]. Decisional conflict was measured using the Decisional Conflict Scale (DCS), a tool for the measurement of healthcare consumer decision uncertainty, factors contributing to uncertainty, and healthcare consumer perceived effective decision-making [13]. The DCS uses items derived from the decisional conflict construct: uncertainty, selected factors contributing to uncertainty, and perceptions of effective decision-making. Participants were asked to reflect on the specific treatment decisions made and to indicate the extent to which they agreed or disagreed with the statements by providing a number from 1 (“strongly agree”) to 5 (“strongly disagree”). In accordance with the instrument’s user manual, we defined DCS sum scores exceeding 37.5 with decision delay or feeling unsure about implementation. DCS scores lower than 25 were associated with implementing decisions.

To measure psychological distress, we used the National Comprehensive Cancer Network (NCCN) Distress Thermometer. This screening instrument assesses psychosocial stress in oncological patients on a scale from 0, meaning “no distress”, to 10, meaning “maximum distress” [14]. Internationally, a cutoff value of ≥ 5 is recommended as a signal that a patient is noticeably distressed and requires support.

Finally, we assessed objective knowledge with five knowledge questions from the “Decision Quality Worksheet: For Treating Prostate Cancer” [15]. The questionnaire was designed to be administered during decision-making or after a decision has been made. The five questions measure the extent to which patients are informed, and we implemented the German translation in 2016 [7, 9]. The five questions were as follows:

- 1. **Without treatment**, about how many men diagnosed with early-stage prostate cancer will eventually die of prostate cancer?
- A Most will die of prostate cancer.
- B About half will die of prostate cancer.
- C Most will die of something else [correct answer].
- 2. For most men with early-stage prostate cancer, how much would **waiting a few months** to make a treatment decision hurt their chances of survival?
- A A lot.
- B Somewhat.
- C A little or not at all [correct answer].
- 3. In the first few years after treatment for prostate cancer, which is **more** likely to cause bowel problems?
- A Surgery.
- B Radiation [correct answer].
- C Both surgery and radiation are equally likely to cause bowel problems.
- 4. In the first few years after treatment for prostate cancer, which is more likely to **cause sexual problems with erections**?
- A Surgery.
- B Radiation.
- C Both surgery and radiation are equally likely to cause sexual problems [correct answer].
- 5. In the first few years after treatment for prostate cancer, which is more likely to cause **dripping or leaking urine**?
- A Surgery [correct answer].
- B Radiation.
- C Both surgery and radiation are equally likely to cause dripping or leaking urine.

### Ethics

This study was conducted in accordance with the latest version of the Declaration of Helsinki. Data collection was part of the postmarket surveillance for a medical device, and we analyzed only anonymous data. Therefore, no ethics vote was required for this project.

### Statistics

We included only cases without any missing data within the three core outcomes (DCS score, distress, and knowledge). For categorical data, we report relative frequencies, and for metric data, we report the means and the standard deviations as a measure of dispersion. Group comparisons were made using the chi-square test for nominal variables and the paired t test for quantitative data (two-sided, significance level α = 0.05). All calculations were performed using SPSS 29.0 (IBM, Armonk, NY, USA).

## Results

During the nine-month study period, 2,245 patients used the PC decision aid. Complete data were available for 880 patients (39.2%). The mean patient age was 66.3 ± 7.2 (range 43–84) years. The oncological risk groups were low in 20.1%, intermediate in 52.4%, and high in 26.5% of the patients. Metastatic disease was reported by 0.6% of the patients. The majority of the patients (62.3%; 548/880) preferred collaborative decision-making, 33.6% (296/880) preferred an active role, and 4.1% (36/880) preferred a passive role. Patient sociodemographic data are compared to those of a previously reported large sample in Table 1. While many variables were consistent, users with complete data in the current evaluation had higher education levels (A level: 41.4% vs. 36.1%; p < 0.001) and higher incomes (> €4,000: 27.4% vs. 22.4%; p < 0.001).

**Table 1:** Patient sociodemographic data (N = 880) compared with a previously reported large sample (N = 11,290)

Variable		Routine use N = 11,290	Valid percentages	Study cohort N = 880	Valid percentages	p
*Age*						0.1
	≤ 50	212	1.9%	9	1.0%	
	51–60	2,344	20.8%	197	22.4%	
	61–70	5,214	46.3%	421	47.9%	
	71–80	3,307	29.4%	235	26.7%	
	> 80	182	1.6%	17	1.9%	
	Missing	31	n/a	1	n/a	
*Marital status*						0.5
	Firm partnership	7,643	89.8%	800	91.0%	
	Single (e.g. divorced, widowed)	788	9.3%	72	8.2%	
	Other	77	0.9%	7	0.8%	
	Missing	2,782	n/a	1	n/a	
*Children*						0.6
	Yes	7,302	86.1%	747	85.4%	
	No	1,179	13.9%	128	14.6%	
	Missing	2,809	n/a	5	n/a	
*German language skills*						0.3
	Mother tongue	7,818	91.8%	821	93.4%	
	Fluent	638	7.5%	53	6.0%	
	Low	60	0.7%	5	0.6%	
	Missing	2,774	n/a	1	n/a	
*Education*						< 0.001
	A-Levels	3,064	36.1%	364	41.4%	
	Medium	2,545	30.0%	302	34.4%	
	Low	2,215	26.1%	167	19.0%	
	Other	672	7.9%	46	5.2%	
	Missing	2,794	n/a	1	n/a	
*Place of residence*						0.7
	< 10.000 inhabitants	3,041	35.7%	320	36.4%	
	> 10.000 inhabitants	5,467	64.3%	559	63.6%	
	Missing	2,782	n/a	1	n/a	
*Health insurance*						0.09
	Public	6,310	74.2%	662	75.2%	
	Private	2,103	24.7%	215	24.4%	
	Other	96	1.1%	3	0.3%	
	Missing	2,781	n/a	0	n/a	
*Income*						< 0.001
	< 1.500 €	478	5.6%	36	4.4%	
	1.500–4.000 €	4,813	56.8%	420	50.9%	
	> 4.000 €	1,900	22.4%	226	27.4%	
	No information provided	1,284	15.2%	143	17.3%	
	Missing	2,815	n/a	55	n/a	

All three main outcomes are summarized in Table 2.

**Table 2:** Summary of the main outcomes: decision conflict, distress and knowledge (sum score), paired t test

Outcome	Prior	Post	p
Decision conflict scale (total)	37.5 ± 23.7	20.1 ± 15.7	< 0.001
Decision conflict scale (level of information)	3.7 ± 2.8	1.4 ± 1.6	< 0.001
Decision conflict scale (clarity)	3.6 ± 2.9	1.6 ± 1.6	< 0.001
Decision conflict scale (support)	2.7 ± 2.2	1.7 ± 1.6	< 0.001
Decision conflict scale (uncertainty)	4.8 ± 3.0	2.9 ± 2.4	< 0.001
Distress	6.2 ± 2.6	3.1 ± 2.6	< 0.001
Knowledge (sum score)	2.3 ± 1.1	3.0 ± 1.1	< 0.001

### Decisional conflict

**Fig. 1:**
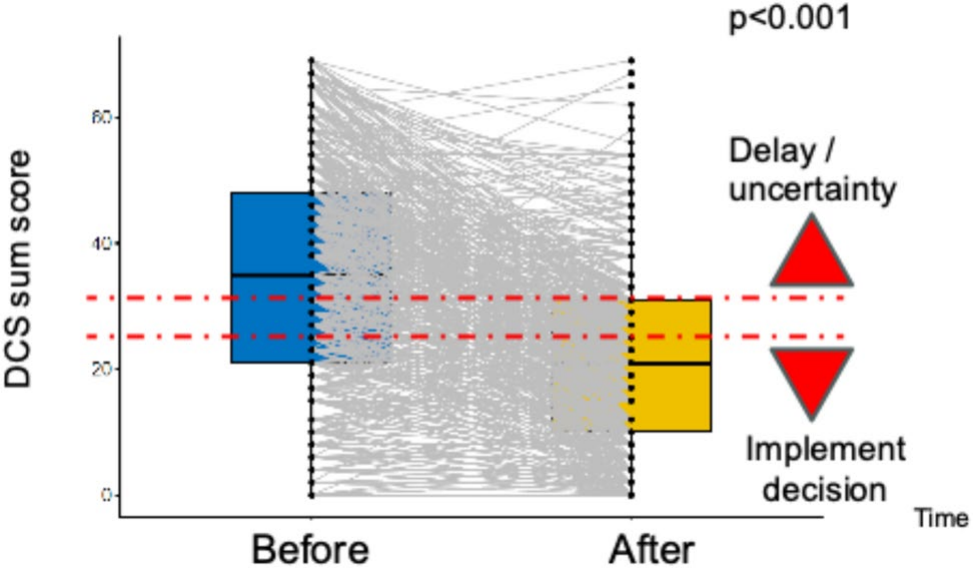
Decisional conflict: Spaghetti diagram with a decrease across both predefined cutoff values

The DCS score improved from 37.5 ± 23.7 before to 20.1 ± 15.7 after the decision aid was used (p < 0.001). Figure 1 depicts the corresponding spaghetti diagram, which illustrates the decline in decisional conflict from greater than 37.5 for a state of uncertainty to under the level of 25 for implementing a decision. These observations were consistent for all the DCS subscales: the levels of information (3.7 ± 2.8 vs. 1.4 ± 1.6), clarity (3.6 ± 2.9 vs. 1.6 ± 1.6), support (2.7 ± 2.2 vs. 1.7 ± 1.6), and uncertainty (4.8 ± 3.0 vs. 2.9 ± 2.4) decreased after the use of the PC decision aid (all p < 0.001).

### Distress

**Fig. 2:**
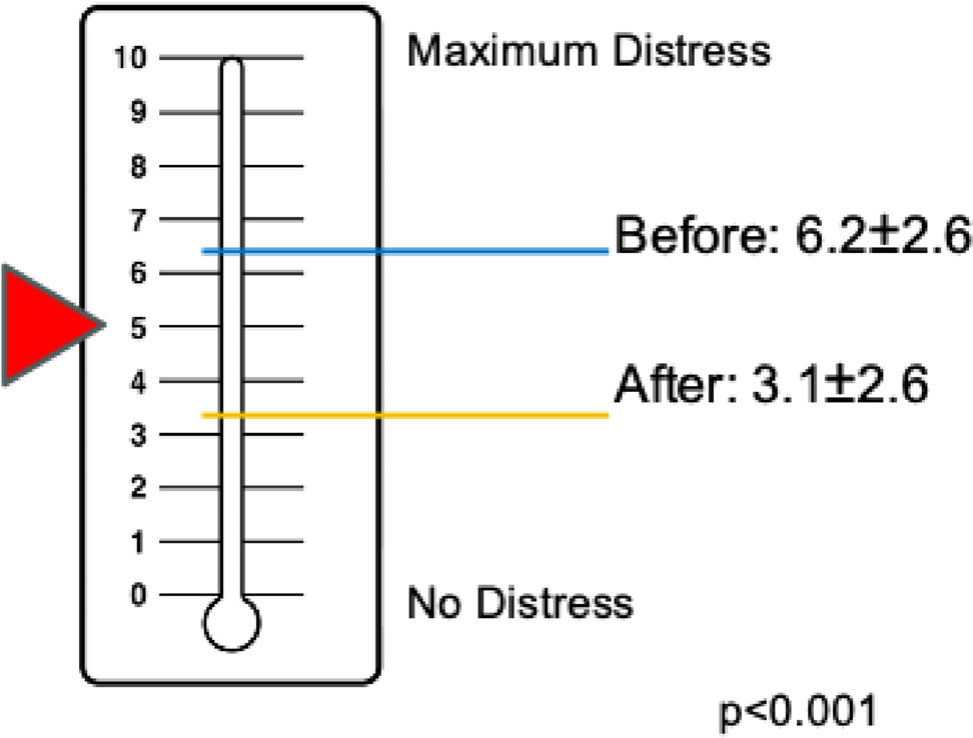
Distress before and after using the PC decision aid

The Distress Thermometer (0: no distress, to 10: maximum distress) showed an improvement of 3.1 points from 6.2 ± 2.6 to 3.1 ± 2.6 (p < 0.001). Figure 2 illustrates the decline in the distress from above the cutoff value of ≥ 5 to below using the visual analog scale of the Distress Thermometer.

### Knowledge

**Fig. 3:**
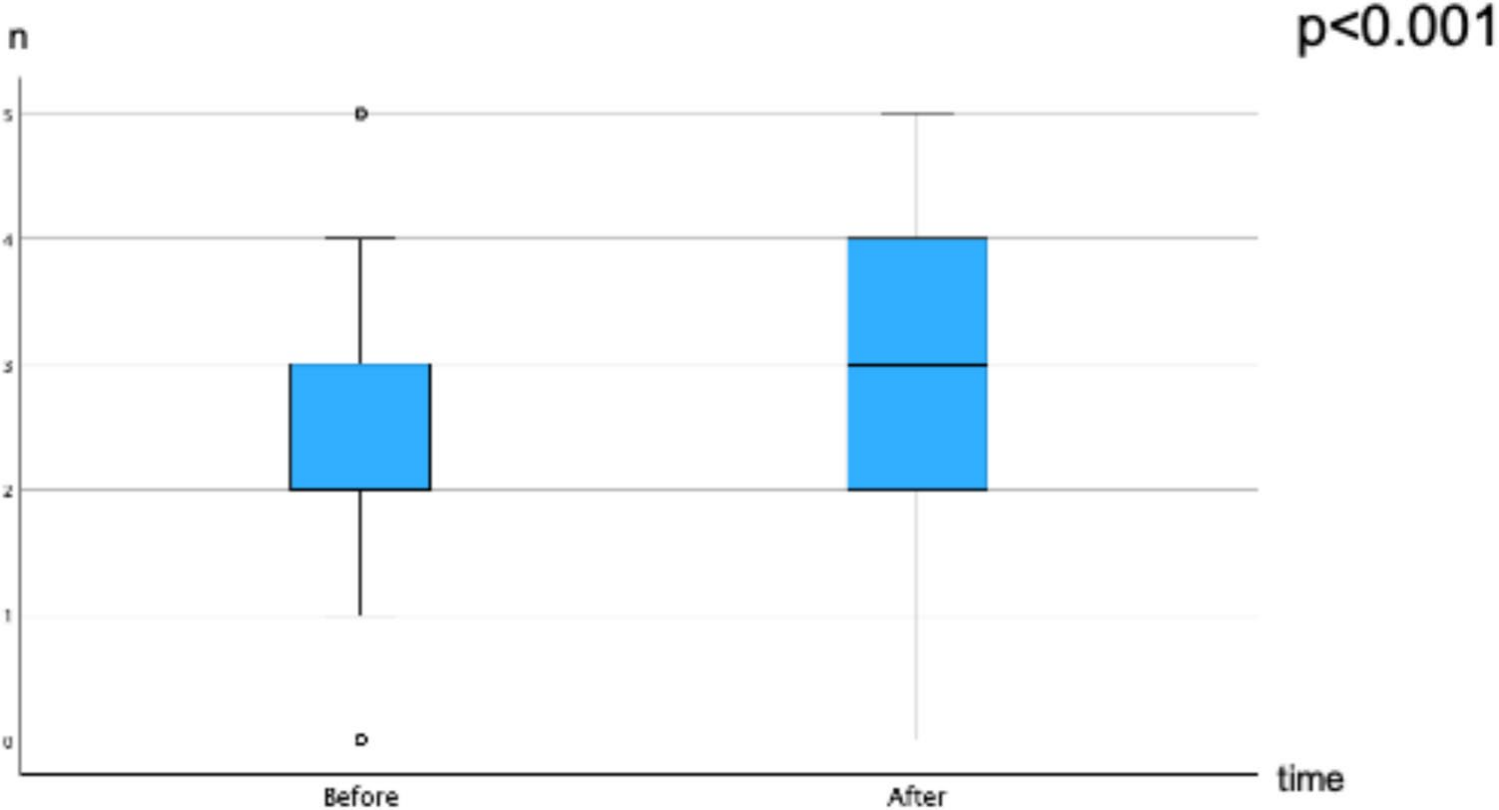
Knowledge test: number of correct answers to five questions before and after the use of the PC decision aid

Objective knowledge significantly improved in 4 out of the 5 questions, with differences in absolute percentages of correct answers ranging from 18.8% to 30%. The percentages of correct answers before vs. after using the PC decision aid were as follows: question 1 regarding cancer-specific mortality, 32.3% vs. 62.3%; question 2 regarding treatment delay, 23.2% vs. 43.0%; question 3 regarding bowel toxicity, 55.0% vs. 73.8%; and question 4 regarding erectile dysfunction, 61.6% vs. 83.3% (p < 0.001 each). Only question 5 regarding urinary incontinence had a lower percentage of correct answers after the use of the PC decision aid (59.2% vs. 33.8%; p < 0.001). In terms of the correct number of answers before vs. after using the PC decision aid, there was a significant increase of 2.3 ± 1.1 vs. 3.0 ± 1.1 (p < 0.001), respectively. Figure 3 shows the overall results of the knowledge test.

## Discussion

Our postmarket surveillance in German routine care shows intraindividual improvements in patient decisional conflict, distress, and knowledge after an online PC decision aid is used. These improvements are clinically relevant because they exceed the predefined limits of validated questionnaires measuring levels of decisional conflict and distress. Moreover, there were significant improvements related to 4 out of the 5 knowledge questions, and the sum of correct answers increased significantly.

### Decisional conflict

Decisional conflict is among the best-established concepts in shared decision-making research [6]. The DCS score and all the subscale scores improved after the use of the PC decision aid. This change might be associated with a greater likelihood of implementing a reasonable decision. Several studies show that prostate cancer decision aids reduce decisional conflict at a substantial and clinically relevant degree [16–19]. A RCT in men recently diagnosed with prostate cancer used a decision aid versus usual information. Within the study Decisional Conflict Scale (0–100) dropped from 53.0 ± 16.9 to 31.2 ± 10.2 in the intervention group, whereas the control group remained essentially unchanged (49.1 ± 13.7 to 51.7 ± 13.3; P < 0.001) [20].​ This 22‑point absolute reduction versus control is generally considered a large, clearly clinically relevant change on the DCS. Another RCT (n = 122) compared brochure-only education with software-based preference assessment plus brochure.​ Baseline mean DCS was 2.3 ± 0.9 on a 1–5 scale (moderate conflict). The preference-assessment decision aid arm had a significantly larger decrease in total DCS score (P = 0.023) and in the Perceived Effective Decision Making subscale (P = 0.003) compared with education alone [21].

### Distress

Using the PC decision aid also helped affected patients significantly reduce their distress. This emphasizes that high-quality, patient-oriented treatment information improves the psychological impact. Moreover, the recorded distress value is also included in the physician summary and enables automated screening for the need for psycho-oncological support. The effects of decision aids on distress played only a subordinate role in previous studies and showed little to no difference [6]. A systematic review on PC decision aids did not address distress and revealed no effects on decisional regret or preparedness in decision‐making [4]. Future research results from a randomized controlled trial of an online treatment decision aid for men with low-risk prostate cancer and their partners are pending [5, 22].

### Knowledge

Knowledge about a disease and possible treatment-associated side effects is another important criterion for being able to adequately take part in shared decision-making. After using the PC decision aid, patients had better knowledge about cancer-specific mortality, the safety of delaying active treatment, and treatment-associated side effects on bowel toxicity and erectile function. Only the fifth knowledge question was answered more incorrectly after the use of the PC decision aid. Within the provided educational videos, the difference in relative frequencies of severe urinary continence is broken down to 9 of 100 patients after surgery vs. 4–6 of 100 patients after radiation therapy [23]. This difference may not be perceived as relevant by the patients, and a majority reported that “both surgery and radiation are equally likely to cause dripping or leaking urine”. Given that surgery causes higher rates of urinary incontinence, we will discuss this special aspect within the next update.

### Limitations and strengths

We are aware of several limitations: owing to the use of anonymous data, we were unable to perform an additional follow-up, and it was not possible to add further clinical data. All reported outcomes are derived from intra-individual comparisons, which are susceptible to distortion. This might impose a possible bias, especially for the measurement of objective knowledge. Patient attention is driven to the content of these questions, which might improve their gain of knowledge. Moreover, some patients might also use additional sources to answer certain items correctly. However, we believe this effect to be small because the number of correct answers in a large population without prior access to the questions was 2.9 ± 1.1 [7] and almost equals the current number of 3.0 ± 1.1. As the analysed sample with complete data had higher education levels and higher incomes, the observed effects on decisional conflict could be biased. Initial decisional conflict could be lower or the effect of the tool could be more pronounced because of better health literacy [24]. The present study population had a higher percentage of better educated patients with higher incomes [7]. As socioeconomic status can have a significant effect on the usage and reception of health-related information, this induces further possible bias. To overcome these basic limitations, we launched a randomized controlled study to test these results by comparing a group without decision aid use and a group in which the PC decision aid was used.

The post-DA use data was assessed immediately upon termination of use of the DA because Decisional conflict is only valid during the decision process. This is important, because the retrospective assessment of decisional conflict weeks or even month after a decision was assessed, biases its assessment.

The strengths of the current work are the large recent population from routine care and the application of validated endpoints. The results are an ideal complement to the EvEnt-PCA study to critically evaluate the role of the survey time points [9]. Although strong and clinically relevant effects can be observed immediately after using the PC decision aid, these effects subside after four weeks. Nine years after its implementation, our PC decision aid is an established standard for providing PC treatment information in Germany. Therefore, our project may serve as an example for successfully implementing decision aids in routine clinical practice [4].

### DiGA

Since the Digital Healthcare Act was passed in Germany in 2019, digital health applications (DiGA = Digitale Gesundheitsanwendungen) have increasingly found their way into the healthcare market [25]. DiGAs are low-risk digital medical devices (e.g., apps and browser-based applications) that can be prescribed at the expense of public insurance if their effectiveness has been shown [26, 27]. They offer great potential for development in the transformation and modernization of healthcare and pose major challenges for the evolving digital healthcare landscape [26–29]. However, DiGAs are rarely embedded in existing care structures, and the benefits are often unclear at the outset. As a result, doctors still rarely prescribe DiGAs. However, for a DiGA to offer real added value for patient care and to be recommended to suitable target groups as part of patient empowerment, for example, they must fulfill specific evidence and quality criteria [30–32].

Currently, there are three DiGAs for treating urological disorders. Kranus Edera was the first urological DiGA to be permanently listed for the treatment of erectile dysfunction and its causes [33]. As a second urological DiGA, Kranus Lutera was recently registered as a digital therapy for men with lower urinary tract symptoms (LUTS) [34]. Third, Uroletics provides pelvic floor exercises for PC patients before or after radical prostatectomy [34, 35]. In this context, our online PC decision aid plays a special role, as there is currently no DiGA that supports shared decision-making.

## Conclusion

In conjunction with the results of our previous work [9], we can now report strong immediate effects that diminish over a period of four weeks: the evaluated online PC decision aid alleviates decisional conflict and distress and improves patient knowledge. These results provide further support for the use of all suitable patients for this purpose. An ongoing randomized controlled study will reduce the current study limitations.

## Supplementary Information

Below is the link to the electronic supplementary material.

Supplementary file1 (MP4 298097 kb)

## Data Availability

No datasets were generated or analysed during the current study.

## Abbreviations

BPS: German Federal Association for Prostate Cancer Self-Help/Bundesverband Prostatakrebs Selbsthilfe e.V.
DCS: Decisional conflict scale
DiGA: Digital health application/Digitale Gesundheitsanwendung
DGU: German Society of Urology/Deutsche Gesellschaft für Urologie e.V.
MDD: Medical device directive
NCCN: National comprehensive cancer network
PC: Prostate cancer
PHQ: Patient Health questionnaire
USG: Urological foundation health gGmbH/Urologische Stiftung Gesundheit gGmbH
